# Comparative analyses of the *Sox9a*-*Amh*-*Cyp19a1a* regulatory Cascade in Autotetraploid fish and its diploid parent

**DOI:** 10.1186/s12863-020-00840-8

**Published:** 2020-03-21

**Authors:** Xu Huang, Qinbo Qin, Kaijun Gong, Chang Wu, Yuwei Zhou, Qian Chen, Wenjing Feng, Yiying Xing, Chongqing Wang, Yude Wang, Liu Cao, Min Tao, Shaojun Liu

**Affiliations:** State Key Laboratory of Developmental Biology of Freshwater Fish, College of Life Sciences, Hunan Normal University, Changsha, 410081 Hunan PR China

**Keywords:** Autopolyploidization, *Sox9a*-*Amh*-*Cyp19a1a*, DNA methylation, Genetic/epigenetic diversity

## Abstract

**Background:**

Autotetraploid *Carassius auratus* (4nRCC, 4n = 200, RRRR) was derived from the whole genome duplication of diploid red crucian carp (*Carassius auratus* red var.) (2nRCC, 2n = 100, RR). To investigate the genetic effects of tetraploidization, we analyzed DNA variation, epigenetic modification and gene expression changes in the *Sox9a*-*Amh*-*Cyp19a1a* regulatory cascade between 4nRCC and 2nRCC.

**Results:**

We found that the *Sox9a* gene contained two variants in 2nRCC and four variants in 4nRCC. Compared with that in 2nRCC, DNA methylation in the promoter regions of the *Amh* and *Cyp19a1a* genes in 4nRCC was altered by single nucleotide polymorphism (SNP) mutations, which resulted in the insertions and deletions of CpG sites, and the methylation levels of the *Sox9a*, *Amh* and *Cyp19a1a* genes increased after tetraploidization. The gene expression level of the *Sox9a*-*Amh*-*Cyp19a1a* regulatory cascade was downregulated in 4nRCC compared with that in 2nRCC.

**Conclusion:**

The above results demonstrate that tetraploidization leads to significant changes in the genome, epigenetic modification and gene expression in the *Sox9a*-*Amh*-*Cyp19a1a* regulatory cascade; these findings increase the extant knowledge regarding the effects of polyploidization.

## Background

Fertile polyploids play important roles in promoting the exchange of genetic material among species, enriching species diversity, and laying foundations for polyploid breeding [1–4]. Polyploidy can be classified into autopolyploidy and allopolyploidy [5, 6]. The former can be produced by the whole genome duplication (WGD) of a species (autopolyploidization), whereas the latter refers to the merging of genomes from different species after hybridization (allopolyploidization) [7]. Autopolyploidization is of great significance in the origin and evolution of vertebrates; however, it is rare in teleost fishes [8–12]. In previous studies, fertile allotetraploids (4nRB, 4n = 148) were successfully obtained in the first generation derived from the distant hybridization of *Carassius auratus* red var. (2nRCC, 2n = 100, female) × *Megalobrama amblycephala* (BSB, 2n = 48, male) [13]. Fertile autotetraploids (4nRCC, 4n = 200) were obtained in the second generation through crossing diploid sperm and diploid eggs produced by special chromosome behavior in germ cells of 4nRB [14]. The 4nRCC possessed four sets of chromosomes derived from 2nRCC, which produced diploid gametes [14, 15]. We successfully established the autotetraploid lineage (F_2_-F_13_) through continuous self-crossing. Phenotypic changes occurred both in 4nRB and 4nRCC, including the presence of the gray body color and the barbel, which were absent in 2nRCC [14]. Furthermore, the morphological traits of 4nRCC obviously differed from those of 4nRB (e.g. lateral scales, upper lateral scales, dorsal fins, and anal fins) [14]. Hence, this autotetraploid fish lineage provides not only abundant diploid gamete resources for polyploid genetic breeding but also an excellent model to study the consequences of inheritance and evolution in polyploid vertebrates.

The *Sox9*-*Amh*-*Cyp19a1* regulatory cascade was identified in 2013, which played a role in sex differentiation and maintenance in Atlantic cod [16]. This regulatory cascade had a similar role in the gonad development of zebrafish [17, 18]. In this regulatory cascade, *Sox9* (SRY-box containing gene 9) activated the expression of *Amh* (anti-Müllerian hormone), which induced the degeneration of the Müllerian ducts and inhibited the expression of *Cyp19a1* (cytochrome P450 family 19 subfamily A member 1) [19–21]. Testicular expression of *Sox9a* in adult Atlantic cod and zebrafish was consistent with male-specific expression of *Sox9* in mammal [16–18]. *Amh* had functions in both sexes in fish, especially in males, in which it was the basic factor underlying sexual differentiation [22]. In addition, P450 aromatase (*Cyp19a1a*), a key steroidogenic enzyme that converted androgen into estrogen, was widely accepted to be actively involved in ovarian formation, development, and maintenance in fish [23, 24]. Although the *Sox9a*-*Amh*-*Cyp19ala* regulatory cascade played a crucial role in the complex process of sex differentiation in some vertebrates, studies of conserved regulatory elements in 2nRCC and 4nRCC could be served as the starting point for a broader understanding of the impact of tetraploidization processes.

The autotetraploid fish lineage provides good experimental material for the study of genomic DNA variation in polyploids, which often exhibited changes in gene structure, gene expression and DNA methylation [25–30]. To investigate the genetic effects of tetraploidization, we analyzed DNA variation, epigenetic modification and gene expression changes in the *Sox9a*-*Amh*-*Cyp19a1a* regulatory cascade between 4nRCC and 2nRCC. This is the first report concerning the *Sox9a*-*Amh*-*Cyp19a1a* regulatory cascade in autotetraploid fish, and its results provide a broad survey of the effects of polyploidization.

## Results

### Cloning and homology analysis of CDS regions

**Fig. 1:**
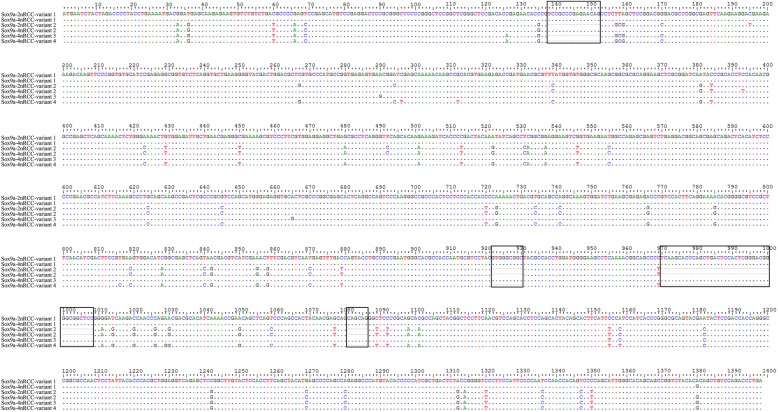
Alignment of the *Sox9a* variant sequences of 2nRCC and 4nRCC. Deletions and insertions in all sequences are framed by black boxes. Dots represent sequence identity

**Fig. 2:**
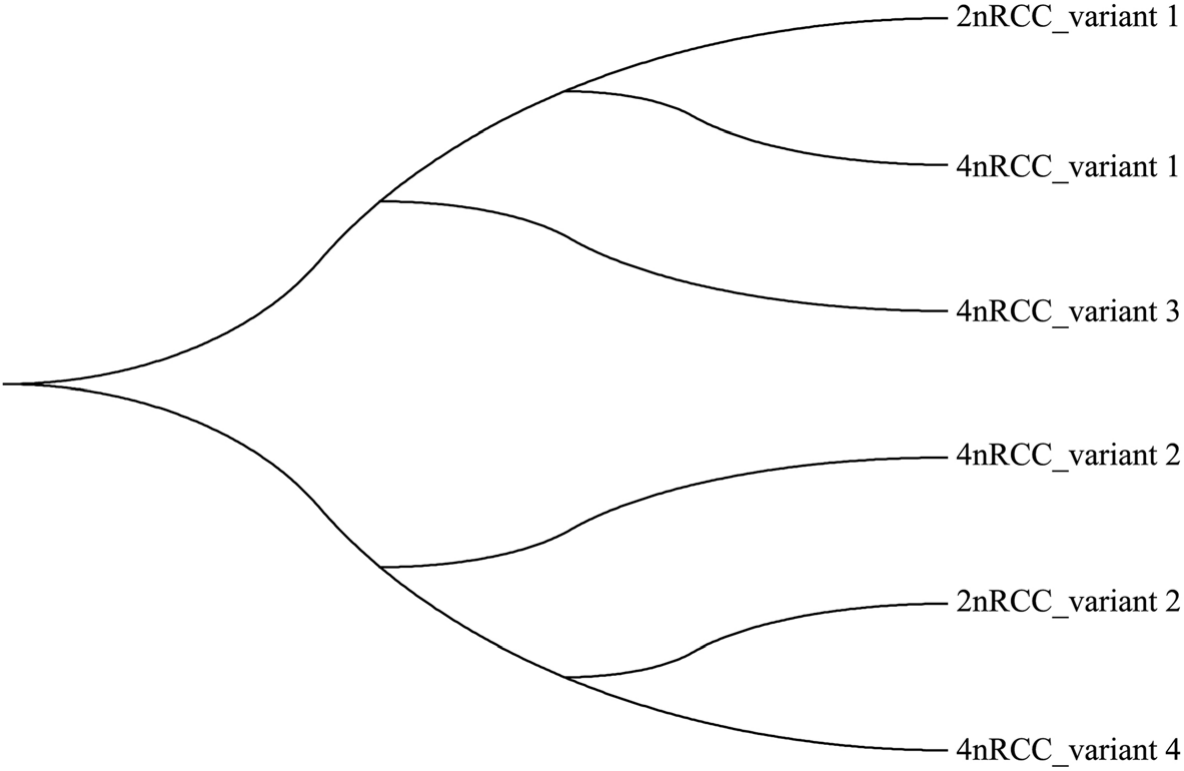
Phylogenetic analysis demonstrating the relationships among the *Sox9a* variant sequences of 2nRCC and 4nRCC using the neighbor-joining method in MEGA7

By splicing the intermediate fragments and 3′ RACE sequences of *Sox9a, Amh,* and *Cyp19a1a*, we obtained complete CDS regions. For the *Sox9a* gene, two different forms in 2nRCC (GenBank Accession Nos. MK307791 and MK307773) and four different forms in 4nRCC (GenBank Accession Nos. MK307774-MK307777) with high homology were observed (namely, variants 1 and 2 in 2nRCC, variants 1 to 4 in 4nRCC); the sizes of the CDS region of these genes were 1398, 1329, 1398, 1344, 1383 and 1329 bp, respectively. For the *Amh* gene, the complete CDS lengths in 2nRCC and 4nRCC were 1707 bp and 1695 bp (GenBank Accession Nos. MK224515 and MK224514), respectively. The complete CDS length of *Cyp19a1a* in both 2nRCC and 4nRCC was 1557 bp (GenBank Accession Nos. MK224513 and MK224512). Homology analysis showed that 2nRCC and 4nRCC had high homology at the amino acid sequence level, with amino acid identities of 90% for *Amh* and 98% for *Cyp19a1a*. As shown in Fig. 1, CDS alterations of *Sox9a* involving SNP insertions, deletions and mutations were observed. Phylogenetic analysis was used to determine the relationships among the *Sox9a* variant sequences in 2nRCC and 4nRCC using the neighbor-joining method in the MEGA7 software (Fig. 2).

### Expression of *Sox9a*, *Amh*, and *Cyp19a1a* in mature gonads

**Fig. 3:**
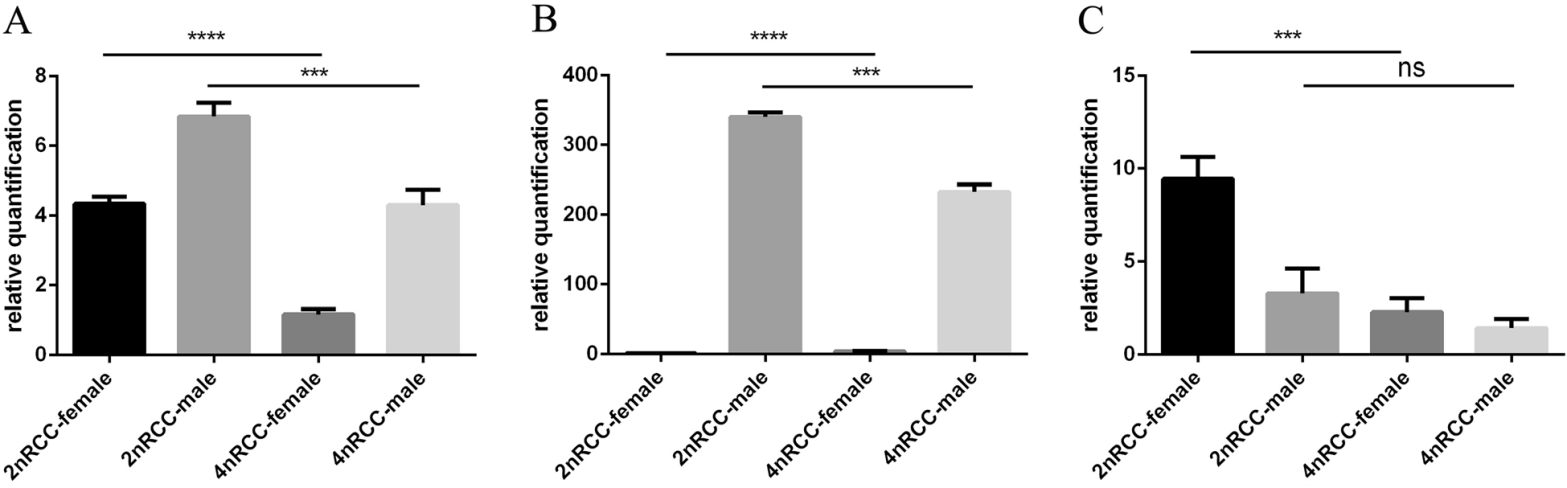
Relative quantification of *Sox9a* (**a**), *Amh* (**b**), and *Cyp19a1a* (**c**) in 2nRCC and 4nRCC. Both *** and **** indicate significant differences between 2nRCC and 4nRCC (*P* < 0.01); ns indicates no significant difference between 2nRCC and 4nRCC

To investigate the expression of the target genes in mature gonads, qPCR was used to compare expression patterns between 2nRCC and 4nRCC. The results showed that the expression of *Sox9a* and *Amh* was predominant in the mature testes (Fig. 3a and b; *P* < 0.01), whereas the expression level of *Cyp19a1a* was higher in the mature ovaries (Fig. 3c; *P* < 0.01). Compared with 2nRCC, 4nRCC showed downregulated expression levels of *Sox9a* in the mature testes and ovaries (Fig. 3a; *P* < 0.01), and the expression pattern of *Amh* was observed to be same as that of *Sox9a* (Fig. 3b; *P* < 0.01). As shown in Fig. 3c, in the mature ovaries, 2nRCC showed high expression of *Cyp19a1a* compared with that of 4nRCC (*P* < 0.01), but there was no significant difference in the mature testes (*P* > 0.05). These results showed that the expression of the target genes in the *Sox9a*-*Amh*-*Cyp19a1a* regulatory cascade was down-regulation following polyploidization.

### DNA methylation status of the *Sox9a*, *Amh*, and *Cyp19a1a* genes

**Fig. 4:**
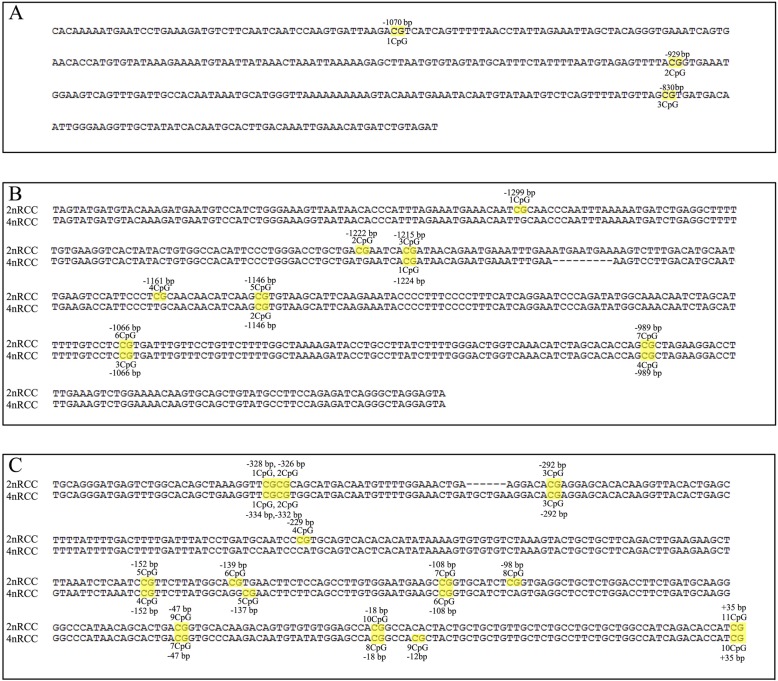
Amplified regions of the predicted promoter sequences relative to the transcription start site. Numbers with a plus or minus sign show CpG positions relative to the transcription start site. The yellow boxes represent CpG dinucleotide sites in the target gene promoter. **a** Three CpG sites in the *Sox9a* gene promoters of 2nRCC and 4nRCC are marked with yellow boxes. **b** Seven CpG sites in the *Amh* gene promoter of 2nRCC are marked with yellow boxes, whereas only four CpG sites are present in the 4nRCC promoter. **c** Ten CpG sites in the *Cyp19a1a* gene promoter of 2nRCC are marked with yellow boxes, and nine CpG sites in the promoter of 4nRCC are marked. The last CpG site in both 4nRCC and 2nRCC was not included due to the primer position

**Fig. 5:**
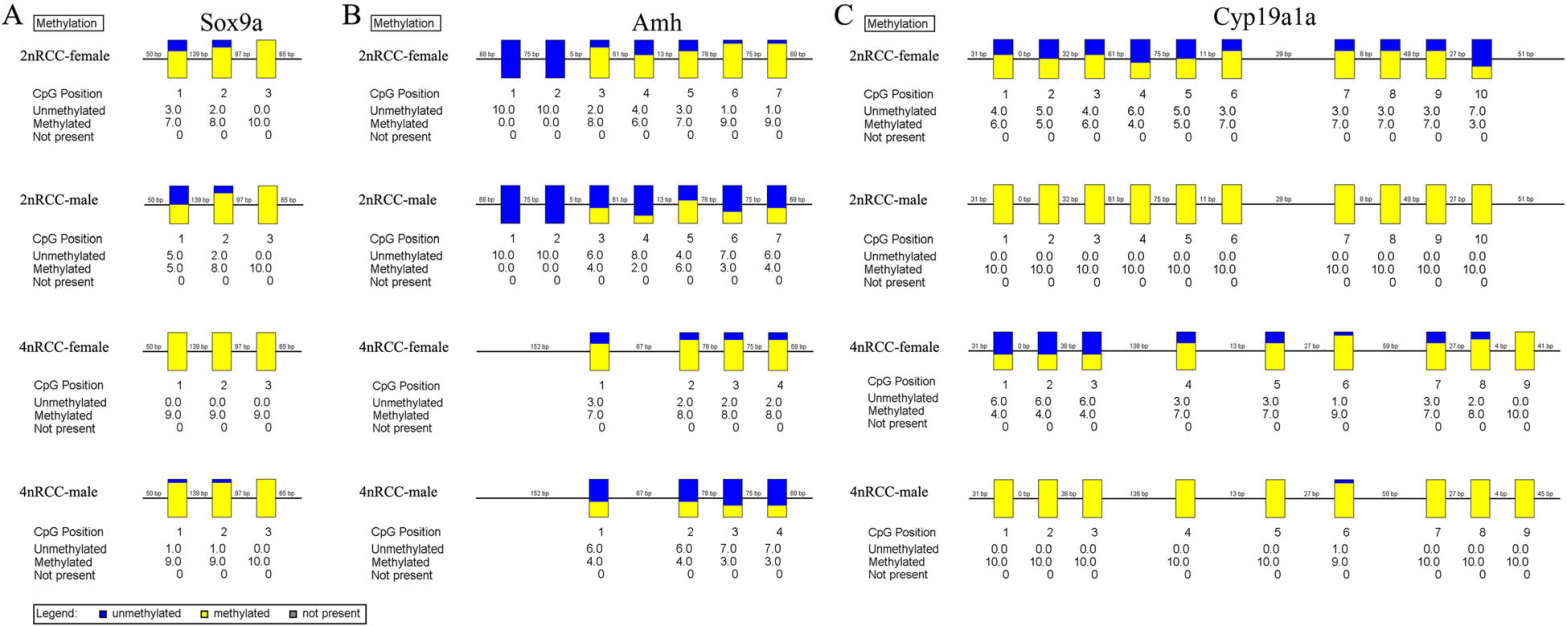
Differential CpG methylation status of *Sox9a* (**a**), *Amh* (**b**) and *Cyp19a1a* (**c**) promoters in mature testes and ovaries of 2nRCC and 4nRCC. Each box corresponds to one CpG position in the genomic sequence. Ten clones per sample were analyzed. Yellow boxes indicate methylation status, blue boxes indicate unmethylated status, and gray boxes indicate the non-present CpG positions

**Fig. 6:**
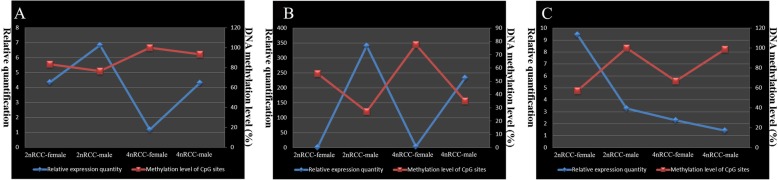
Correlation between the expression of the *Sox9a* (**a**), *Amh* (**b**) and *Cyp19a1a* (**c**) genes and CpG methylation in 2nRCC and 4nRCC

Using bisulfite sequencing and BiQ Analyzer software, we evaluated the differential DNA methylation status of target gene promoters in the *Sox9a*-*Amh*-*Cyp19a1a* cascade in the mature gonads of 2nRCC and 4nRCC. Among the 3 CpGs found between − 1120 bp and − 764 bp of the *Sox9a* promoter sequence (Fig. 4a), the average methylation levels of 4nRCC were higher than those of 2nRCC in the mature testes and ovaries (Fig. 5). Numbers with a plus or minus sign show CpG positions relative to the transcription start site (http://www.fruitfly.org/seq_tools/promoter.html). Compared with the 2nRCC sequence, the 4nRCC *Amh* gene had deletions of three CpG sites, and the *Cyp19a1a* gene had three deleted CpG sites and two newly inserted CpG sites (Fig. 5). In 2nRCC and 4nRCC, 7 CpGs at − 1363 bp and − 915 bp, 4 CpGs at − 1372 bp and − 915 bp were found in the *Amh* promoter sequence, respectively (Fig. 4b). In addition, 10 CpGs at − 359 bp and + 36 bp, 9 CpGs at − 365 bp and + 36 bp were found in the *Cyp19a1a* promoter sequences of 2nRCC and 4nRCC, respectively (Fig. 4c). These results revealed that SNP mutations resulted in the insertions and deletions of CpG sites. We demonstrated the average methylation levels (Table 1) and differential CpG methylation status (Fig. 5) of the *Sox9a*, *Amh* and *Cyp19a1a* promoters in the mature testes and ovaries of 2nRCC and 4nRCC. As shown in Fig. 6, the methylation levels of the target gene promoters showed an obvious negative correlation with gene expression levels.

**Table 1:** Average methylation levels of all CpG sites in 2nRCC and 4nRCC

Gene name	Ovary-2nRCC	Testis-2nRCC	Ovary-4nRCC	Testis-4nRCC
*Sox9a*	83.3%	76.7%	100%	93.3%
*Amh*	55.7%	27.1%	77.5%	35%
*Cyp19a1a*	57%	100%	66.7%	98.9%

## Discussion

After genome duplication, polyploids were thought to display changes in their genome structure, gene expression, and epigenetic modification [1, 28, 31]. The structural changes to the genome in polyploids consisted of deletions, insertions, duplications, translocations and transpositions [32]. Some duplicated genes might undergo pseudogenization, neofunctionalization or subfunctionalization during polyploidization [16, 33]. In previous studies, obvious DNA variation, pseudogenization and sequence divergence were observed in allotetraploids and autotetraploids [33, 34]. Although 4nRCC was originated from 2nRCC, the CDS regions and amino acid sequences of *Sox9a* gene presented large differences, which provided direct evidence of genome duplication and alterations following tetraploidization. First, the newly synthetic polyploidy required to solve the problem of survival and reproductive fitness, meaning they needed to overcome the genomic incompatibility and transcriptome shock by genomic changes. Importantly, a variety of genomic alterations at structural level (e.g. extensive intra- and interchromosomal rearrangements ensuring normal chromosome pairing in meiosis) had been shown to re-establish the genome balance after WGDs [10]. Thus, we speculated that the genomic alteration was used to adapt the tetraploidization, which not only increased genomic flexibility and the rate of gene differentiation but also had great advantages for long-term adaptation and persistence of autopolyploids.

Genetic redundancy and intergenomic interactions were among the general features of polyploids that induced genetic and epigenetic changes (e.g. epigenetic remodeling to silence parts of the duplicated genome), leading to recombination of the genome and regulatory networks [10, 35]. Compared with that of 2nRCC, the methylation level of the *Sox9a*-*Amh*-*Cyp19a1a* regulatory cascade increased in 4nRCC after genome duplication, and a similar result was found previously in the autopolyploid *Chrysanthemum lavandulifolium* [6]. Meanwhile, the genomic alterations in the promoter region of target genes led to insertions and deletions of CpG sites after an autopolyploidization event, producing an important effect on the genomic methylation status. It is possible that genetic and epigenetic alterations may be related to each other after a polyploidization event. Further, epigenetic changes may regulate the level of gene expression, which had the potential to add the stability of the genome after polyploidization. Notably, the same sex-specific methylation patterns were retained in 4nRCC and 2nRCC. In the previous study, DNA methylation may be a mechanism used for natural sex maintenance, as in the Chinese sea perch *Lateolabrax maculatus*, and a potential regulatory mechanism during temperature-induced sex change, as in Nile tilapia and European sea bass (*Dicentrarchus labrax*) [36–38]. Although the *Sox9a*, *Amh* and *Cyp19a1a* genes presented significant sex correlations in fishes, further experiments are required to verify whether the *Sox9a*-*Amh*-*Cyp19a1a* regulatory cascade is related to sex determination or sex differentiation in 4nRCC and 2nRCC. The results so far suggest that 2nRCC and 4nRCC may have similar sex differentiation mechanisms.

Similarly, to re-establish the transcriptional balance, the changes of gene expression patterns (increase or decrease) were required across the genome [10]. Previous studies of gene expression changes in polyploids had highlighted multiple potential mechanisms, including homologous gene silencing, altered regulatory networks, DNA methylation, and chromatin remodeling [26, 27, 39]. Because allopolyploidy encompasses different genomes and increases genome complexity, autopolyploids are thought to be the best material to evaluate the possibility of ploidy-dependent regulatory changes [26]. Here, the expression levels of the *Sox9a*, *Amh*, and *Cyp19a1a* genes were significantly downregulated (*P* < 0.01) after tetraploidization, which suggested that tetraploidization has the potential to lead to changes in gene expression through the combined effects of genomic variation and DNA methylation. Autopolyploids were generally expected to maintain genomes highly similar to those of their diploid parents and not exhibited significant changes in gene expression [28, 40], whereas our results clearly exhibited such changes. These changes in gene expression may be very important to help polyploids adapt to the consequences of WGD and maintain genome balance.

## Conclusion

Our data demonstrated that tetraploidization was associated with significant changes in genome sequences, epigenetic modifications and gene expression by surveying the *Sox9a*-*Amh*-*Cyp19a1a* regulatory cascade. This study provides important evidence on genomic DNA variation and new information about the effects of polyploidization.

## Methods

### Materials

Both 2nRCC and 4nRCC used in this study were cultivated in ponds with expanded feed at the Protection Station of Polyploid Fish of Hunan Normal University, Hunan, China. During the reproductive season (April–June), both 2nRCC and 4nRCC reached sexual maturity at 1 year-old and produced a large number of mature eggs and white sperm normally, respectively. All fish used as samples were anesthetized with 100 mg/L MS-222 (Sigma-Aldrich, St Louis, MO, USA) prior to dissection. Gonads were excised from three adult males and three adult females under sterile conditions, frozen quickly in liquid nitrogen, and stored at − 80 °C for further use.

### RNA isolation and RT-PCR

Total RNA was isolated from the mature gonad tissues of 2nRCC and 4nRCC using RNAiso reagent (TaKaRa, Japan) following the manufacturer’s protocol. The RNA purity and concentration were measured using Synergy 2 Multi-Mode Microplate Reader (BioTek, www.biotek.com), and their integrity were evaluated using 1% agarose gel electrophoresis [34]. The first-strand cDNA was synthesized using the Maxima H Minus First Strand cDNA Synthesis Kit with dsDNase (Thermo Scientific, USA) in a 20 μl reaction volume. The synthesized cDNA was stored at − 20 °C for further use.

### Coding sequence (CDS) cloning of *Sox9a*, *Amh* and *Cyp19a1a*

Based on the cDNA sequences of the *Carassius auratus* × *Cyprinus carpio* × *Carassius cuvieri Sox9a* gene (DQ201318), the goldfish *Amh* gene (KF640083) and the *Carassius auratus Cyp19a1a* gene (KC147009) available at NCBI, special primers for each gene were designed using Primer Premier 5 software to amplify the intermediate fragments. Subsequently, 3′ RACE was performed with forward primers designed from each intermediate fragment and a reverse adaptor primer. The 3′ RACE products of the target genes from 2nRCC and 4nRCC were sequenced, and their complete CDS regions were obtained after splicing the partial CDS regions. All primers were presented in Table 2, and the PCR products were cloned as described in [41] and sequenced by Tsingke (Beijing, China).

**Table 2:** Nucleotide sequences of primers used for CDS cloning, 3′ RACE and RT-qPCR in this study

Primer name	Nucleotide sequence (5′ to 3′)
For CDS cloning	
*Sox9a*-F1	ATGAATCTACTAGACCCCTACCT
*Sox9a*-R1	GCTGCTGTGCCCAATGCTG
*Amh*-F1	ATGCTCTTCCACGCAGGATTTTG
*Amh*-R1	CAAAGAGCAAAAGGAGGGTGTCA
*Cyp19ala*-F1	ATGGCAGGTGAACTTCTCCAGCC
*Cyp19ala*-R1	GAGGGCGTTTCTGGGGATGAGC
For 3′ RACE	
*Sox9a*-3′-F	CAACTCCTATTACACCCACGCT
*Amh* − 3′-F	CAACAACCACGCCATCCTC
*Cyp19ala* − 3′-F	TGCTCAAACAGAATCCAGAC
3′-adaptor Primer	CTGATCTAGAGGTACCGGATCC
For RT-qPCR	
*β-actin*-F	CATCTACGAGGGTTACGCCC
*β-actin*-R	AATTTCCCTCTCGGCTGTGG
*Sox9a*-F2	TCAATACCCGCACCTCCACAACGCC
*Sox9a*-R2	TGAAGATGGCGTTCGGGGAGATGTG
*Amh*-F2	GGAATTCACCAGTCCTGATAGC
*Amh*-R2	CTGCAGAAGTTCTTGAGTGACG
*Cyp19ala*-F2	TTGACACCTGGCAGACGGTA
*Cyp19ala*-R2	CTGCGATTATCATCTCCAACAC

*CDS* coding sequence, *RACE* rapid amplification of cDNA ends, *RT-qPCR* real-time quantitative polymerase chain reaction

### Quantitative real-time PCR analysis

The cDNAs prepared from the gonads of 2nRCC and 4nRCC were used to detect relative gene expression using quantitative real-time PCR (qPCR). Species-specific primers for a housekeeping gene (*β-actin*) and three target genes (*Sox9a*, *Amh* and *Cyp19a1a*) were designed with Primer Premier 5 software and presented in Table 2. The cDNAs from the adult testes and ovaries of 2nRCC and 4nRCC were used as templates. All of the experiments in this stage were performed three samples and three replicas to improve the accuracy of the results. The qPCR amplification, using ABI Prism 7500 Sequence Detection System (Applied Biosystems, USA), was performed in a 10 μl reaction mixture containing 5 μl of SYBR Green PCR Master Mix (ABI), 1 μl of cDNAs (in a dilution of 1:15), 0.4 μl of each primer (10 μM) and 3.2 μl of sterilized water. The amplification conditions were 50 °C for 5 min, 95 °C for 10 min, and 40 cycles at 95 °C for 15 s and 60 °C for 45 s. After that, the melting curve was performed at the end of the assay to verify the generation of a single product. The dissociation conditions were 95 °C for 1 min, 60 °C for 30 s and 95 °C for 30 s. The relative expression was determined with GraphPad Prism 6 software using the 2^-ΔΔCT^ method [42].

### Genomic DNA isolation and DNA bisulfite modification

Genomic DNA was extracted from all samples of adult testes and ovaries using the TaKaRa MiniBEST Universal Genomic DNA Extraction Kit Ver. 5.0 (TaKaRa, Japan). The methods for detecting DNA purity and concentration were similar to those used after RNA isolation. Then, 400 ng of each sample was sodium bisulfite-modified using the EZ DNA Methylation-Gold™ Kit (Zymo Research, USA) according to the manufacturer’s protocol. The bisulfite-treated DNA was stored at − 20 °C.

### Amplifying promoter regions and predicting CpG-rich regions

The first exon of each of the three target genes was aligned to the genome of 2nRCC (NCBI project accession no. PRJNA289059) using Blast (V2.5.0) software, and the promoter positions were considered to be within 2 kb upstream of the first exon. The promoters of *Sox9a*, *Amh* and *Cyp19a1a* were amplified using untreated genomic DNA as the template, and the primers were listed in Table 3. The CpG-rich regions of the *Sox9a*, *Amh* and *Cyp19a1a* promoters and exons were predicted by the online MethPrimer design software (http://www.urogene.org/index.html).

**Table 3:** Nucleotide sequences of primers used for promoter amplification and BS-PCR in this study

Primer name	Nucleotide sequence (5′ to 3′)
For promoter amplification	
*Sox9a*-P1	GACTCAGAGGAAAGCCAAGC
*Sox9a*-C1	TCTTGAAGTCTGCGAGGCGG
*Amh*-P1	GTTGGAAACCTTGGCTGTCT
*Amh*-C1	GTCGCCACAATCAGCAACAG
*Cyp19ala*-P1	TTAGCGATGAAAGTGGGCGT
*Cyp19ala*-C1	TGTCCGATGGTGTCTGATGG
For BS-PCR	
*Sox9a*-F3	TATAAAAATGAATTTTGAAAGATGT
*Sox9a*-R3	ATCTACAAATCATATTTCAATTTATC
*Amh*-F3	TAGTATGATGTATAAAGATGAATGT
*Amh*-R3	TACTCCTAGCCCTGATCTCTGGA
*Cyp19ala*-F3	ACAACACCTCAAATAAACCCTAC
*Cyp19ala*-R3	CAAAAAAACCTCACTAAAATACACC

*BS-PCR* Bisulfite PCR

### Bisulfite PCR (BS-PCR) and analysis

BS-PCR primers were designed with Primer Premier 5 software based on the sense strand of the bisulfite-modified DNA (Table 3). PCR was performed in a final volume of 50 μl using LA Taq™ (TaKaRa). The amplification conditions were as follows: 4 min at 94 °C; 35 cycles of 30 s at 94 °C, 30 s at 56 °C for *Sox9a*/ *Cyp19a1a* or 54 °C for *Amh*, and 30 s at 72 °C; and 5 min at 72 °C for extension [43]. All PCR products were detected and separated on a 2% agarose gel, purified using the Gel Extraction Kit (Tsingke), cloned into the pMD18-T vector (TaKaRa) and transferred into *E. coli* DH5α (Sangon, China). To investigate the status of each methylation site, at least 10 positive clones obtained after screening by PCR amplification were sequenced by Tsingke (Beijing, China).

### Statistical analysis

Analyses of variance and pairwise comparisons of the data were analyzed by SPSS 17.0 software.

## Data Availability

The datasets supporting the conclusions of this article were available in the GenBank repository with access No. MK307791, MK307773-MK307777, and MK224512-MK224515.

## Abbreviations

4nRCC: Autotetraploid *Carassius auratus*
2nRCC: Diploid red crucian carp (*Carassius auratus* red var.)
SNP: Single nucleotide polymorphism
CDS: Coding sequence
RACE: Rapid amplification of cDNA ends
RT-qPCR: Real-time quantitative polymerase chain reaction
BS-PCR: Bisulfite polymerase chain reaction
BSB: *Megalobrama amblycephala*
4nRB: Allotetraploid *Carassius auratus*
